# A New Tool for Rapid Assessment of Acute Exercise-Induced Fatigue

**DOI:** 10.3389/fnhum.2022.856432

**Published:** 2022-03-16

**Authors:** Yao Lu, Ziyang Yuan, Jiaping Chen, Zeyi Wang, Zhandong Liu, Yanjue Wu, Donglin Zhan, Qingbao Zhao, Mofei Pei, Minhao Xie

**Affiliations:** ^1^Department of Neurology, Medical Health Center, Beijing Friendship Hospital, Capital Medical University, Beijing, China; ^2^Department of Clinical Medicine, Capital Medical University, Beijing, China; ^3^Beijing Peirong Biotech Co., Ltd, Beijing, China; ^4^Daxing No.1 Middle School Beijing, Beijing, China; ^5^Department of Physical Education, Beijing Institute of Petrochemical Technology, Beijing, China; ^6^Baoding No.17 Middle School, Baoding, China; ^7^China Institute of Sports Medicine, Beijing, China

**Keywords:** acute exercise-induced fatigue scale, central fatigue, peripheral fatigue, fatigue assessment, exploratory factor analysis (EFA)

## Abstract

**Background:**

There are limited sensitive evaluation methods to distinguish people’s symptoms of peripheral fatigue and central fatigue simultaneously. The purpose of this study is to identify and evaluate them after acute exercise with a simple and practical scale.

**Methods:**

The initial scale was built through a literature review, experts and athlete population survey, and a small sample pre-survey. Randomly selected 1,506 students were evaluated with the initial scale after exercise. Subjective fatigue self-assessments (SFSA) were completed at the same time.

**Results:**

The Acute Exercise-Induced Fatigue Scale (AEIFS) was determined after performing a factor analysis. In the exploratory factor analysis, the cumulative variance contribution rate was 65.464%. The factor loadings of the total 8 questions were 0.661–0.816. In the confirmatory factor analysis, χ^2^/df = 2.529, GFI = 0.985, AGFI = 0.967, NFI = 0.982, IFI = 0.989, CFI = 0.989, and RMSEA = 0.048. The Cronbach’s alpha coefficient for the scale was 0.872, and it was 0.833 for peripheral fatigue and 0.818 for central fatigue. The intra-class correlation coefficient for the scale was 0.536, and the intra-class correlation coefficients for peripheral fatigue and central fatigue were 0.421 and 0.548, respectively. The correlation coefficient between the total score of the AEIFS and the SFSA score was 0.592 (*p* < 0.01).

**Conclusion:**

Our results demonstrate that the AEIFS can distinguish peripheral fatigue and central fatigue and can also reflect their correlation. This scale can be a useful evaluation tool not only for measuring fatigue after acute exercise but also for guiding reasonable exercise, choosing objective testing indicators, and preventing sports injuries resulting from acute exercise-induced fatigue.

## Background

Fatigue has been a new focus of health-related studies in recent years, since it is a common accompanying symptom of many diseases (Druce and Basu, 2019; Janssen et al., 2020; Siciliano et al., 2020), especially in diseases like myalgic encephalomyelitis/chronic fatigue syndrome (ME/CFS) (Jason et al., 2012; Manca et al., 2021). Exercise-induced fatigue (EF) has always been an important issue in the field of sports medicine (Steele et al., 2017); when the exercise exceeds a certain limit, it increases the possibility of injury, extends the recovery period from fatigue, and prevents athletes from performing well (Bresciani et al., 2011; Verschueren et al., 2020). For non-athletes, acute exercise-induced fatigue (AEF) may also cause injuries and affect their quality of life.

Fatigue originations can be classified as either peripheral or central (Meeusen, 2014; Carroll et al., 2017; Tan et al., 2018; Ratka et al., 2020). Dysfunction of muscle contraction during exercise can induce peripheral fatigue (O’Leary et al., 2016), mainly caused by consumption of energy materials, accumulation of metabolites, and material imbalance in the body (Chang and Zhang, 2006). In comparison, exercise-induced central fatigue is a protective mechanism of the central nervous system manifested as changes in thinking and consciousness to stop exercising (Zhang, 2008). It is often accompanied by symptoms such as headaches, nausea, vomiting, and post-exercise cognitive impairment (Ratka et al., 2020), which may be mediated by brain hypoxia (Siebenmann and Rasmussen, 2016), cortical activity (Shibuya et al., 2004), neurotransmitter changes (Liu et al., 2017), and neuron connection changes (Tan et al., 2018).

Currently, the use of animal model studies has revealed many fatigue mechanisms, especially for central fatigue studies (Gomez-Merino et al., 2001; Cotel et al., 2013; Liu et al., 2017). There are limited sensitive evaluation methods to distinguish people’s symptoms of peripheral fatigue and central fatigue simultaneously (Laurent et al., 2020). Therefore, establishing a simple, effective, and non-invasive assessment of AEF is important to guiding exercise types and intensity, not only in athletes but also in general people, especially in fatigue-related patients.

This study has designed a concise and practical AEF Scale to assess the peripheral and central AEF status quickly and effectively. Furthermore, our scale could be used with the current methods for fatigue assessment, which may help guide reasonable exercise regimens, prevent sports injuries, and promote further selection of special objective test indicators.

## Materials and Methods

### Scale Formulation

#### Question Pool Establishment

Following discussions with sports experts and athletes, we combined a literature review and analysis of fatigue-related studies (Carroll et al., 2017; Tan et al., 2018; Ratka et al., 2020) to determine the main research subject of the 2 types of fatigue. Comprehensively considering previous fatigue-related scales and the physiological characteristics of AEF, 14 questions were initially screened. Among these, 6 questions were based on existing research (Bowman et al., 2004), in addition to 3 questions on physiological indicators such as respiratory rate. The remainder of the questions were formulated specifically for this study.

Each question implemented a 5-point Likert scale (Likert, 1932), and all questions were scored in a positive manner for the description of fatigue, with 0 indicating the mildest fatigue and 4 indicating the most severe fatigue. Examples of question composition are shown in Table 1.

**TABLE 1:** Example of certain items of acute exercise-induced fatigue.

Item	A	B	C	D	E
T1: How difficult is it for you to continue the exercise?	Not at all	A little bit	Moderately	Quite a bit	Extremely
T2: How much do you feel sick and want to vomit now?	Not at all	A little bit	Moderately	Quite a bit	Extremely
T5: How is your current sense of direction compared with the situation before the exercise?	Clearer than before	The same as before	A little unclear	Unclear	Totally lost

*Scoring criteria: For each item, there are 5 choices, namely, A, B, C, D, and E, representing 0, 1, 2, 3, and 4 points, respectively.*

To make the scale questions more accurate and concise, 3 neurology chief physicians, 3 professional coaches, and 2 sports medicine experts were invited to discuss modifications of the preliminary scale, and then, 11 items of questions were selected for the final assessment of the further pre-survey.

#### Small Sample Pre-survey

A total of 280 students from Daxing No.1 Middle School Beijing were selected for a small sample pre-survey. Unsuitable questions were inevitable and were, therefore, manually adjusted based on the number of students and the number of questions completed. Specifically, one question in the scale had to be deleted due to the poor quality of data collection. Ten items of questions were kept in this pre-survey.

#### Students

Participants in the main study were from 5 middle schools and universities in Beijing City and Hebei Province. The inclusion criteria consisted of healthy students studying at school. The exclusion criteria consisted of (1) students who were suffering from diseases, (2) students who were prone to fatigue according to their personal history or not suited to exercise, and (3) students who were unwilling to participate in the study. The assessment method was an 800 m run. To ensure safety, the students were required to try their best to complete the run as quickly as possible. The AEF Scale assessment was conducted immediately after the run. According to their own evaluations, Students’ subjective fatigue was measured by selecting “mild,” “moderate,” or “severe.” The scale was distributed and collected in 15 min at the same spot, and attention was paid to the confidentiality of Students’ responses. The study protocol was approved by the Ethics Committee of Beijing Friendship Hospital, Capital Medical University (approval no. BJFH-EC/2019-P2-034-01).

#### Scale Verification

**FIGURE 1:**
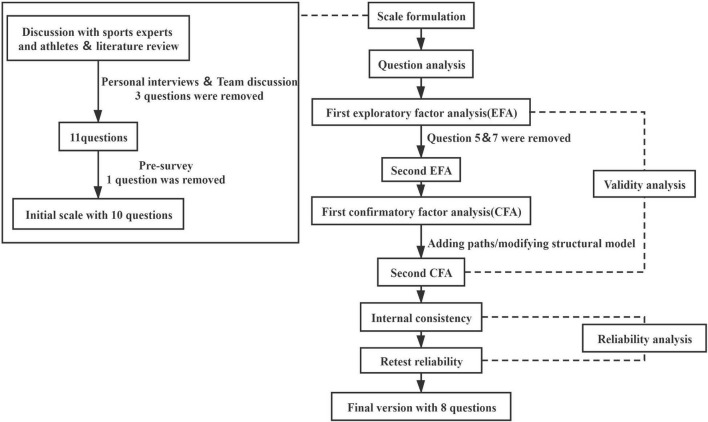
Steps and analysis methods used in the scale formulation.

After the questionnaire was collected, 2 staff members inputted the data, and the original data were sorted and archived to ensure accuracy. The SPSS Statistics for Windows, Version 25.0 (IBM, Armonk, NY, United States) was used to further analyze the data. After adjusting the questions, the final version of the AEF Scale was developed (Figure 1). Question analysis was conducted using the Shapiro-Wilk test.

In the content validity analysis, the exploratory factor analysis (EFA) was performed. EFA is widely used in psychology and behavioral science. EFA examines the underlying structure of a group of variables when the relationships among these variables are unclear (McNeish, 2017). To verify the applicability of the factor structure in another sample population, the confirmatory factor analysis (CFA) was executed with SPSS AMOS for Windows, Version 22.0 (IBM, Armonk, NY, United States). The results of CFA measure how well the correlations observed in the data fit with the correlations predicted by the structural equation model (Chung and Morris, 2015). The internal consistency of reliability reflects the stability and reliability of the scale, which can be evaluated by Cronbach’s α coefficient (Pallant, 2010; DeVellis, 2016) and split-half reliability (Bujang et al., 2018) (the larger the Cronbach’s α coefficient, the higher the internal consistency and the better the homogeneity). Of the students who completed the first scale assessment, 20 cases were randomly selected for retesting at 2-week intervals, and the intragroup correlation coefficient (ICC) was used to reflect the retesting reliability.

## Results

### Baseline Information

Using the ten items, we considered any questionnaire to be invalid when there was any fatigue question/item incomplete of the 10 during the scale assessment. A total of 1,506 questionnaires were collected, and 152 invalid questionnaires were removed from the analysis. Therefore, there were 1,354 valid questionnaires, with an effective response rate of 89.91%. Among the valid questionnaires, 744 (54.95%) questionnaires were filled in by male students and 610 (45.05%) questionnaires were filled in by female students. The ages of the students ranged from 13 to 21 years, with an average age of 16.55 ± 1.65 years. After the first assessment, additional 20 students were randomly selected for retesting after 2 weeks. The average age of the retested students was 15.95 ± 2.40 years, of which 45% were male students and 55% were female students.

### Question Analysis

After the Shapiro-Wilk test analysis, the distribution of scores for each question (10 questions in total) on the AEF Scale was non-parametric (*p* < 0.05). The scores of each question were added to calculate the total score of each Student’s AEF Scale, and the Spearman’s correlation coefficient was used to calculate the correlation between each question and the total score (see Table 2).

**TABLE 2:** Correlation between each item and the total score.

Items	Spearman’s correlation coefficient
T1	0.770**
T2	0.683**
T3	0.631**
T4	0.798**
T5	0.636**
T6	0.658**
T7	0.766**
T8	0.671**
T9	0.748**
T10	0.657**

**Significance: p < 0.01.

The results showed that the Spearman’s correlation coefficient of each question was greater than 0.60, and each question was significantly associated with the total score of the scale (*p* < 0.01), indicating that the questions of the scale are moderately homogeneous (Schober et al., 2018). In terms of distinction degree, through the extreme groups approach (EGA) (Preacher, 2015; Cusimano et al., 2020), the students were divided into a high score group (top 27% of total scores) and a low score group (bottom 27% of total scores) according to their total score. Through a non-parametric Mann–Whitney test analysis of the 2 independent samples, the scores of each student from the 2 groups were shown to be significantly different (*p* < 0.05), suggesting a good distinction between the AEF Scale and the assessment of the fatigue levels.

### Validity Analysis

#### Content Validity

Currently, there is no gold standard to determine the degree of AEF. To ensure the content validity of the AEF Scale, this study consulted 8 experts in related fields during the scale’s creation, and the scale was revised based on the experts’ experience.

#### Exploratory Factor Analysis

The students were randomly divided into 2 groups, and EFA was performed using the data from the first group (*N* = 677). First, Kaiser–Meyer–Olkin (KMO) test and the Bartlett sphere test (Tobias and Carlson, 1969; Kaiser, 1974) were performed to determine whether this set of data was suitable for factor analysis. The KMO value was 0.924, and the Bartlett test of sphericity was significant (*p* < 0.001). Therefore, according to the standard of KMO > 0.9 and *p* ≤ 0.01, the scale data were suitable for factor analysis. After this, the common factors were extracted by principal component analysis, and the common factor method was used for rotation. Factor loadings refer to the strength of the relationship between the variables and a common factor (retention criteria: 1 question’s loading of either factor was greater than 0.4 and loading difference of the question between 2 factors was at least greater than 0.2) (Budaev, 2010; Howard, 2015), while the variance contribution rate refers to the proportion of variation caused by a single common factor in the total variation, which can be thought of as the contribution of the factor. The loadings of all questions of the scale are presented in Table 3.

**TABLE 3:** The loadings of exploratory factors.

Questions	First analysis		Second analysis (Final)	
		
	Factor1 Peripheral fatigue	Factor2 Central fatigue	Factor1 Peripheral fatigue	Factor2 Central fatigue
T1	**0.740**	0.328	**0.751**	0.337
T2	**0.682**	0.292	**0.682**	0.309
T3	**0.749**	0.088	**0.762**	0.087
T4	**0.755**	0.343	**0.755**	0.351
T9	**0.649**	0.322	**0.661**	0.308
T6	0.253	**0.790**	0.258	**0.793**
T8	0.236	**0.808**	0.251	**0.815**
T10	0.304	**0.805**	0.307	**0.816**
T5	0.422	0.473	INV	INV
T7	0.649	0.450	INV	INV
Variance contribution rate (%)	51.968%	9.510%	53.665%	11.799%
Cumulative variance contribution rates (%)	51.968%	61.478%	53.665%	65.464%

*Principal component analysis was performed on 677 subjects using the AEF Scale. After the orthogonal rotation, the loadings of exploratory factors, the variance contribution rate, and the cumulative variance contribution rates were calculated. The loading difference between Factor1 (peripheral fatigue) and Factor2 (central fatigue) was invalid (< 0.2). Bold labels the higher scores of the two factors. INV, invalid.*

The cumulative variance contribution rate refers to the proportion of variation caused by all common factors in the total variation (Streiner, 1994). A total of 2 common factors were extracted, and the cumulative variance contribution rate was 61.478%. Questions 5 and 7 were deleted because the loadings for each question were 0.422, 0.473 and 0.649, 0.450, respectively. EFA was performed again on the remaining 8 questions. Ultimately, two common factors were determined, and the cumulative variance contribution rate was 65.464%. The first factor consisted of 5 questions, namely, T1, T2, T3, T4, and T9; and the second factor contained 3 questions, namely, T6, T8, and T10. In addition, in line with the content of the question, the first and second factors represented peripheral fatigue and central fatigue, respectively. Each question was within the originally set dimensions and conformed to the design of the scale structure.

#### Confirmatory Factor Analysis

In this analysis, the second group of students (*N* = 677) was included with “peripheral fatigue” and “central fatigue” as the 2 latent variables of the model. Eight questions served as observed variables for the CFA using a series of indicators such as χ^2^/df to reflect model fit (Byrne, 2013; Goursand et al., 2013; Brown, 2015; Chung and Morris, 2015). The specific fit values of the initial structure model are given in Table 4.

**TABLE 4:** Fitting result of confirmatory factor analysis.

	χ^2^/df	GFI	AGFI	NFI	IFI	CFI	RMSEA
Initial structure model	3.966	0.974	0.950	0.968	0.976	0.976	0.066
Revised structure model	2.529	0.985	0.967	0.982	0.989	0.989	0.048
Recommended values	<3	>0.9	>0.9	Close to 1	Close to 1	Close to 1	<0.05 (good)

*χ^2^/df, chi-square; GFI, Goodness-of-Fit Index; AGFI, Adjusted Goodness-of-Fit Index; NFI, Normal Fit Index; IFI, Incremental Fit Index; CFI, Comparative Fit Index; RMSEA, Root-Mean-Square Error of Approximation.*

As seen in the table, the initial structural model index fitted well referring to the current guidelines (Netemeyer et al., 2003; Hair et al., 2009) and recommended standard (Hooper et al., 2008; Brown, 2015; Kline, 2015). However, with reference to the best-fit upper bound cutoff value of χ^2^/df < 3, the model was further optimized in this study based on the suggested value of the revised Modification Index (MI). After adding paths between e1 and e3 and between e3 and e4, the CFA was performed again.

**FIGURE 2:**
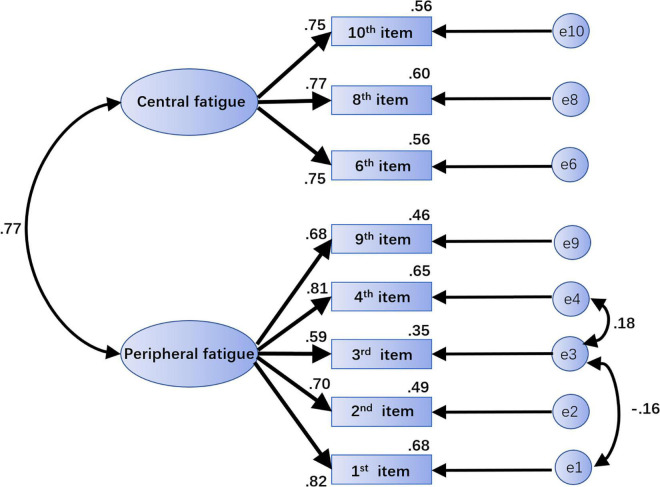
The standardized path coefficient graph of the final structural model. The path “→” indicates influence or causality; the path “↔” indicates correlation or covariance. The number on each path is the standardized regression weight. Central fatigue and peripheral fatigue are latent variables. “e1,” “e2,” “e3,” “e4,” “e9,” “e6,” “e8,” and “e10” are unique variables. Paths can be added between e1 and e3 and between e3 and e4 to modify the model.

All indicators of the modified model reached the optimal adaptation criteria with ideal fitting degree (Netemeyer et al., 2003; Hooper et al., 2008; Hair et al., 2009; Brown, 2015; Kline, 2015). The specific values are shown in Table 4, and the standardized path coefficient of the final structure model is provided (Figure 2). Furthermore, using the correlation analysis method to verify the structural validity of the scale, the correlation coefficient between the 2 factors was 0.625, and the correlation coefficients between peripheral fatigue, central fatigue and total score were 0.959 and 0.809, respectively, indicating a strong correlation.

### Reliability Analysis

#### Internal Consistency

In this study, the total Cronbach’s α coefficient of the AEF Scale was 0.872, and the Cronbach’s α coefficients of the 2 factors of peripheral fatigue and central fatigue were 0.833 and 0.818, respectively, reaching the ideal cutoff values for the internal consistency (Taber, 2017). Furthermore, to assess internal consistency reliability, the average of the correlation between the results of all possible pairs of questions was calculated. From the final 8 questions, 28 pairs were measured, and the average of their correlation was calculated, which was 0.458. The Guttman split-half coefficient was 0.888. All reached the ideal cutoff values (Glen, 2018; Duplaga et al., 2019).

#### Retest Reliability

The retest reliability ICC of the total scale was 0.536, and the retest reliability ICCs of the central fatigue factor and peripheral fatigue factor were 0.548 and 0.421, respectively. According to the ICC standard, the retest reliability of the scale was acceptable (Kottner et al., 2011; Li et al., 2015).

#### Correlation Analysis of Subjective Fatigue Assessment

A total of 1,282 scales with complete subjective fatigue evaluation results were collected, of which 496 showed mild fatigue (38.69%), 675 showed moderate fatigue (52.65%), and 111 showed severe fatigue (8.66%). The evaluation results were based on the positive scoring method, with 1–3 points representing mild, moderate, and severe subjective fatigue awareness, respectively. The Spearman’s correlation coefficient was used to calculate the correlation between the subjective fatigue evaluation score and the total score of the AEF Scale. The peripheral fatigue factor score, central fatigue factor score, and the correlation coefficients were 0.592, 0.604, and 0.409, respectively (*p* < 0.01); all were moderately related (Schober et al., 2018), indicating that the designed scale reflects subjective awareness of fatigue after exercise to a certain extent.

## Discussion

As a common symptom, AEF is important to daily life and sports training. Since AEF is common after exercise, its evaluation can be divided into examining objective and subjective indicators. Objective indicators include heart rate, blood pressure, respiration rates, and biomedical markers (Bresciani et al., 2011; Nelson and Asplund, 2016), with the latter becoming more and more popular (Nunes et al., 2014; Vargas and Marino, 2014). However, the results of some markers, such as blood analysis, cannot be returned quickly (Martinent et al., 2014). Additionally, due to the sophistication of sample collection procedures, serial testing is not appropriate.

This study combined relevant hypotheses, empirical research, and expert opinions to compile the AEF Scale. Additionally, by requiring participants to assess fatigue immediately after the exercise test, recall bias of fatigue awareness caused by rest was minimized. The results of this study indicate that the AEF Scale has good reliability and sensitivity and could be used to quickly assess the fatigue status of students after exercise in order to promptly guide exercise decisions.

In terms of effectiveness, both central and periphery factors were extracted in the EFA. All questions maintained their original dimensions, and all factor loadings were between 0.661 and 0.816, indicating that the AEF Scale had a valid structure (Budaev, 2010). In the CFA, the maximum likelihood method was used to evaluate the model in the scale according to 2 factors, and the results showed good fit indices (Netemeyer et al., 2003; Hooper et al., 2008; Hair et al., 2009; Brown, 2015; Kline, 2015).

Regarding the factor loading results, the cumulative variance contribution rates of peripheral fatigue and central fatigue were 53.665 and 11.799%, respectively, which differed considerably because it took some time to produce central fatigue (Meeusen et al., 2006) following the occurrence of peripheral fatigue. Our research measured the 800 m run (the exercise time was relatively short) that was only used as the exercise stimulation in this experiment. In this test, Students’ fatigue evaluations showed that only 8.66% of students reported severe fatigue, suggesting that most of the Students’ central fatigue might be mild or moderate and leading to a low central fatigue factor variance contribution rate.

In terms of reliability, the internal consistency of the AEF Scale was favorable. The total Cronbach’s α coefficient of the scale was 0.872, and the Cronbach’s α coefficients of the two factors were both greater than the reference value of 0.800 (Taber, 2017). Additionally, the retest reliability ICC of the total scale was 0.536, and the ICCs of the two factors were 0.548 and 0.421, indicating that the retest reliability was consistent (Li et al., 2015). In the question analysis, the scale was well distinguished, and the eight questions that constitute the AEF Scale were significantly correlated with the total score (*r* = 0.631–0.798), which suggested that the questions were related well to the scale (Schober et al., 2018).

The results showed that there was a moderate correlation between the peripheral fatigue factor and central fatigue factor (*r* = 0.625). Although both factors were extracted from EFA, questions 1, 2, 4, and 9, regarding peripheral fatigue, may still be affected by their high loadings of central fatigue (see Table 3); this suggests that these questions might simultaneously contribute to the central fatigue factor. Previous studies have found that certain other factors, such as CO_2_ (Subudhi et al., 2011; Fan et al., 2013), could affect both peripheral fatigue and central fatigue. When exercising for a long time in a high-temperature environment, central fatigue could also mediate peripheral fatigue performance by affecting the maximum voluntary contraction force of skeletal muscles (Taylor and Gandevia, 2008). At the same time, muscle fatigue might also trigger a series of effects that ultimately affect the functions of the central nervous system (such as the neuronal circuitry) (Pollak et al., 2014). Overall, in the development of fatigue, central fatigue and peripheral fatigue might overlap and interact. Therefore, the strong correlation between the two factors in this scale might in fact be a reflection of the characteristics of fatigue itself.

Based on symptoms and relation in our study, an alternation in the sense of direction after exercise is considered as a symptom of central fatigue, which reflects a change of cognitive function in the brain during fatigue and can be separated from peripheral fatigue symptoms. On the contrary, degrees of nausea and vomiting may be caused by multiple reasons, so it is considered as a symptom of peripheral fatigue and might be related with 5-HT metabolism during acute exercise (Cordeiro et al., 2017). A previous study indicated that the peripheral serum level of tryptophan was significantly increased in acute fatigue after exercise (Strasser and Fuchs, 2016), which affected the metabolism of tryptophan and led to nausea and vomiting by increased peripheral, but not central, 5-HT level (Fernstrom and Fernstrom, 2006). Decreased muscle power is another major symptom of fatigue. Some studies showed that central fatigue played an important role (Tanaka and Watanabe, 2012; Jiang et al., 2019; Weavil and Amann, 2019). Our initial scale has a question to reflect the change of muscle power after acute exercise. It was removed from the scale after factor analysis because it was unable to differentiate peripheral and central fatigue.

However, this study had certain limitations. First, only healthy teenagers aged 13–21 years from several schools in northern China were selected. Second, only a single 800 m run was used as the experimental exercise stimulation. As such, the applicability of the scale to different age groups, people with different health conditions, and different sports needs to be verified with a larger sample size.

Among the scale indicators, the retest reliability score demonstrated general consistency. This result might be related to the fact that the students in the reliability retest had to exercise every day. After 2 weeks of physical exercise, the Student’s awareness of fatigue under the same exercise conditions changed, which was also in line with the characteristics of sports medicine and could not be explained simply as the under-optimization of the scale formulation, which required further design of special exercise programs and in-depth research. We did notice that the results of the correlation between the scale and subjective fatigue might be influenced by the degree of fatigue in the participants, which depends on their self-report to be accurate. Thus, there were limitations to scale tools related to scientific issues such as fatigue, revealing the importance and urgency of further developing objective indicators.

## Conclusion

The reliability and validity of the AEF Scale were up to the standard of designing a scale, and the questions were concise. The higher a participant scored on the scale, the severer their degree of AEF. Therefore, the scale can be used to evaluate peripheral fatigue and central fatigue quantitatively with relation to AEF. In addition, the scale could be used as a fatigue quantification tool to execute further research on the degree of fatigue and incidence of exercise-related adverse events and as a quantitative tool for further development and verification of objective AEF indicators.

## Data Availability Statement

The original contributions presented in the study are included in the article/supplementary material, further inquiries can be directed to the corresponding author/s.

## Ethics Statement

The study protocol was reviewed and approved by the Ethics Committee of Beijing Friendship Hospital, Capital Medical University (approval no. BJFH-EC/2019-P2-034-01).

## Author Contributions

ZL, ZY, YL, JC, and ZW designed, conducted the experiments, analyzed the data, and wrote the manuscript. YW and MX designed and wrote the manuscript. DZ, QZ, and MP interpreted the data and oversaw the recruitment of participants. All authors contributed to the article and approved the submitted version.

## Conflict of Interest

YW was employed by Beijing Peirong Biotech Co., Ltd. The funder was not involved in the study design, collection, analysis, interpretation of data, the writing of this article or the decision to submit it for publication. The remaining authors declare that the research was conducted in the absence of any commercial or financial relationships that could be construed as a potential conflict of interest.

## Publisher’s Note

All claims expressed in this article are solely those of the authors and do not necessarily represent those of their affiliated organizations, or those of the publisher, the editors and the reviewers. Any product that may be evaluated in this article, or claim that may be made by its manufacturer, is not guaranteed or endorsed by the publisher.

## Abbreviations

SFSA: subjective fatigue self-assessments
AEIFS: Acute Exercise-Induced Fatigue Scale
ME/CFS: myalgic encephalomyelitis/chronic fatigue syndrome
EF: exercise-induced fatigue
AEF: acute exercise-induced fatigue
EFA: exploratory factor analysis
CFA: confirmatory factor analysis
ICC: intragroup correlation coefficient
INV: invalid
EGA: extreme groups approach
KMO: Kaiser–Meyer–Olkin
χ ^2^/df: chi-square
GFI: Goodness-of-Fit Index
AGFI: Adjusted Goodness-of-Fit Index
NFI: Normal Fit Index
IFI: Incremental Fit Index
CFI: Comparative Fit Index
RMSEA: Root-Mean-Square Error of Approximation
MI: Modification Index.
